# Silencing of long noncoding RNA PVT1 inhibits podocyte damage and apoptosis in diabetic nephropathy by upregulating FOXA1

**DOI:** 10.1038/s12276-019-0259-6

**Published:** 2019-08-01

**Authors:** Dong-Wei Liu, Jia-Hui Zhang, Feng-Xun Liu, Xu-Tong Wang, Shao-Kang Pan, Deng-Ke Jiang, Zi-Hao Zhao, Zhang-Suo Liu

**Affiliations:** 1 Department of Nephrology, The First Affiliated Hospital of Zhengzhou University, 450052 Zhengzhou, China; 2 Research Institute of Nephrology, Zhengzhou University, 450052 Zhengzhou, China; 3 Key Laboratory of Precision Diagnosis and Treatment for Chronic Kidney Disease in Henan Province, 450052 Zhengzhou, China; 4 Core Unit of National Clinical Medical Research Center of Kidney Disease, 450052 Zhengzhou, China

**Keywords:** Molecular biology, Cell biology

## Abstract

The number of patients with diabetic nephropathy (DN) is still on the rise worldwide, and this requires the development of new therapeutic strategies. Recent reports have highlighted genetic factors in the treatment of DN. Herein, we aimed to study the roles of long noncoding RNA (lncRNA) plasmacytoma variant translocation 1 (PVT1) and histone 3 lysine 27 trimethylation (H3K27me3) in DN. A model of DN was established by inducing diabetes in mice with streptozotocin. Mouse podocyte clone 5 (MPC5) podocytes and primary podocytes were cultured in normal and high glucose media to observe cell morphology and to quantify PVT1 expression. The roles of PVT1 and enhancer of zeste homolog 2 (EZH2) were validated via loss-of-function and gain-of-function in vitro experiments to identify the interactions among PVT1, EZH2, and forkhead box A1 (FOXA1). The podocyte damage and apoptosis due to PVT1 and FOXA1 were verified with in vivo experiments. PVT1 was highly expressed in MPC5 and primary podocytes in DN patients and in cultures grown in high glucose medium. A large number of CpG (C-phosphate-G) island sites were predicted at the FOXA1 promoter region, where PVT1 recruited EZH2 to promote the recruitment of H3K27me3. The silencing of PVT1 or the overexpression of FOXA1 relieved the damage and inhibited the apoptosis of podocytes in DN, as was evidenced by the upregulated expression of synaptopodin and podocin, higher expression of Bcl-2, and lower expression of Bax and cleaved caspase-3. The key findings of this study collectively indicate that the suppression of lncRNA PVT1 exerts inhibitory effects on podocyte damage and apoptosis via FOXA1 in DN, which is of clinical significance.

## Diabetic kidney disease: Improving treatment by targeting an RNA molecule

Targeting an RNA molecule responsible for disrupting metabolic protein levels in diabetic kidney disease may improve treatment. Diabetic nephropathy (DN) can affect people with type I or type II diabetes, and results in functional deterioration and the need for regular dialysis. DN incidence is rising worldwide, but existing treatments are only partially effective. Zhang-Suo Liu at Zhengzhou University, China, and co-workers examined the role of a long noncoding RNA molecule known as PVT1, which has been recently associated with kidney disease. The team collected serum samples from 32 patients with DN, and also generated a DN mouse model. They found that PVT1 expression was significantly higher in DN, and that this inhibited the expression of a key metabolic protein, FOXA1. Silencing PVT1 restored FOXA1 levels, limiting damage and cell death in kidney cells.

## Introduction

Diabetic nephropathy (DN), a main cause of end-stage renal disease, occurs in 30–40% of patients who require maintenance dialysis, thus posing more burden on health insurance programs^1^. In addition to the conventional, modifiable risk factors, such as smoking, hyperlipidemia, hypertension, and hemodynamic changes, hereditary components are regarded as potential risk factors for DN^2^. Despite the fact that the pathogenesis of DN has been clarified, the development of effective treatment methods for DN remains a challenging task^3^. It has been reported that the current standard therapy only achieves partial renoprotection, which greatly increases the need for novel and effective therapeutic approaches^4^. Evidence has demonstrated that DN usually occurs in familial clusters, highlighting the great significance of DN-related genetic factors in the development of DN and in the identification of more effective therapeutic strategies for DN^5^.

Long noncoding RNAs (lncRNAs) have lengths >200 nt and can regulate the expression of the target gene, and many lncRNAs have been suggested to participate in the pathogenesis of DN, including plasmacytoma variant translocation 1 (PVT1)^6^. PVT1 is an lncRNA (1.9 kb) that encodes numerous alternative transcripts, but is incapable of producing a protein, and it is the first lncRNA that has been found to be related to kidney disease^7^. Enhancer of zeste homolog 2 (EZH2) has been identified as the catalytic subunit of the chromatin remodeling polycomb-repressive complex 2 (PRC2)^8^. A functional study has reported that EZH2 usually works as an inhibitor of cancer suppressor genes and that lncRNAs can regulate the growth and metastasis of tumor cells by binding to EZH2^9^. Histone 3 lysine 27 trimethylation (H3K27me3) is a transcription-suppressive histone modification whose methylation is achieved via EZH2^10^. One study suggested that the downregulation of EZH2 can decrease H3K27me3 in teratoma-forming cells^11^. Forkhead box A1 (FOXA1), also known as HNF3A, belongs to the forkhead family of DNA-binding proteins, which have been noted for their regulatory roles in metabolism^12^. H3K27me3, EZH2, and DNA methyltransferases (DNMT)1/3a/3b have been documented to regulate the methylation and repression of FOXA1 by interacting with each other such that they can be recruited to the endogenous FOXA1 promoter^13^. Therefore, based on the aforementioned literature, we can hypothesize that lncRNA PVT1 promotes the recruitment of H3K27me3 in the FOXA1 promoter region by recruiting EZH2, thereby reducing the expression of FOXA1. In the present study, we aimed to test this hypothesis, to explore the relationship between PVT1 and DN, and to explore the pathomechanism of PVT1 in causing DN in the hopes of developing a new and effective strategy to treat this disease.

## Materials and methods

### Ethical statement

All the patients signed informed consent, which conformed to the Declaration of Helsinki and was approved by the Ethics Committee of the First Affiliated Hospital of Zhengzhou University. All animal experiments in this study conformed to the principles of management and the use of local laboratory animals.

### Study subjects

A total of 32 patients with early DN treated at the First Affiliated Hospital of Zhengzhou University from April 2015 to April 2016 were selected, and their serum samples were collected. The patients included 19 males and 13 females with an average age of 53.6 ± 7.5 years, ranging from 35 to 70 years; all patients met the World Health Organization (WHO) diagnostic criteria for diabetes^14^. The DN patients had a negative urine protein test, with 30–300 mg/24 h urine albumin, indicating early renal damage. Renal damage caused by other primary and secondary factors or by complications was not found. At the same time, the normal sera of 26 healthy controls were collected; the control group included 16 males and 10 females with an average age of 56.4 ± 3.2 years, ranging from 37 to 70 years, and without high-risk factors for renal damage. The isolated sera were immediately preserved in liquid nitrogen at −80 °C.

### Model establishment

A total of 70 7-week-old male C57BL/6 mice (Laboratory Animal Center of Kunming Medical University, Kunming, Yunnan, China) were housed in the specific pathogen-free animal room with a 12 h day/night cycle with alternating light and dark at 22 ± 2 °C in a relatively humid atmosphere of 55 ± 5%. Among these mice, 10 mice were used as the control group, and 60 were used for the establishment of DN mouse models. The mice were weighed one time every 2 weeks and fed a standard diet for 1 month. When the mice weighed approximately 20 g, their kidneys were removed on the same side. After the operation, the mice used for the establishment of DN models were fed with fodder containing high fat, high sugar, and high cholesterol, while those in the control group continued to be fed the standard diet. Mice in both groups were granted free access to adequate supplies of drinking water. Then, 2 months after the operation, the mice used for the establishment of DN models were given streptozotocin at 55 mg/kg via intraperitoneal injection for 5 consecutive days. The changes in fasting blood glucose levels, urine sugar levels, urine volume, and the 24 h urine microalbumin excretion rate were monitored 72 h after injection. If the following standards were met three consecutive times, then the DN model was considered successfully established: (1) fasting blood glucose >16.6 mmol/L, (2) strongly positive for high levels of urine sugar, (3) urine volume >150% of the primary urine volume, and (4) 24 h urine microalbumin excretion rate >30 rag/24 h. Among the 60 mice used to establish the model, 6 died and 3 failed. Then, 10 mice were randomly selected from the remaining 51 DN mice as the DN group, and the remaining 41 mice were divided into 4 groups with 10 mice in each group: the short hairpin (sh)-negative control (NC) group, sh-PVT1 group, GSK126 (EZH2 inhibitor) group, and overexpressed (OE)-PVT1 + OE-FOXA1 group. The fasting blood glucose level, 24 h urine volume, plasma urea nitrogen, urine protein/creatinine ratio, and kidney weight/body weight ratio of the mice in the four groups were determined at the eighth week after the DN model was established.

### Periodic acid-Schiff staining

The renal tissues were then fixed in a 4% paraformaldehyde solution and sectioned into paraffin sections with a thickness of 4 μm. Then, the sections were treated for 7 min with 0.5% periodic acid for oxidation and washed three times with distilled water (5 min each time). The sections were treated with colorless magenta for 7 min in the dark. After the staining solution was absorbed, the sections were treated with 0.5% sodium sulfite for 1 min and then washed with clean water for 5 min. Subsequently, the sections were stained with hematoxylin for 3 min for nuclear staining, were then washed with clean water, and were differentiated with hydrochloric acid alcohol for several seconds. After the sections were washed with clean water, they were treated with ammonia water for 1 min to return to blue and then washed with clean water. After the sections were dehydrated and cleared two times with dimethylbenzene (5 min each time), they were sealed with neutral gum and dried in the ventilator cabinet. Finally, the sections were observed under an optical microscope.

### Culture of primary podocytes

The mouse glomeruli were separated through the differential screening method. The renal cortex was isolated after kidney extraction in a sterile environment. Then, the kidney was gently ground and filtered through 80, 150, and 200 mesh filters layer by layer. The glomeruli on the 200 mesh filters were collected, inoculated into a cell bottle and then incubated in an incubator with constant humidity and 5% CO_2_ at 37 °C_._ After 4.5 h of incubation, the bottle was turned over and normally placed. Then, 7–8 days later, the cells were cultured for 7 days after the first passage, and the morphology of the podocytes was observed under an electron microscope when the cells settled at the bottom of the bottle. The culture lasted for 7 days in normal glucose (NG) medium until the podocytes were completely differentiated and formed extensive connections with each other. Then, the cells were further cultured in serum-free medium for another 24 h to synchronize the cells in each group. Further culture was conducted for 48 h in the culture media containing NG (GS 5 mmol/L) and high glucose (HG, GS 30 mmol/L).

### Culture of podocytes

The immortalized mouse podocyte cell line mouse podocyte clone 5 (MPC5) was purchased from the Cell Bank of the Chinese Academy of Sciences (Shanghai, China), was removed from the liquid nitrogen, and was immediately placed into a water bath at 37 °C with gentle shaking after the liquid nitrogen had evaporated. When completely melted, the cell cryopreservation-containing cells were transferred to a sterilized 5 mL centrifuge tube and centrifuged for 4 min at 2115 × *g*. After the supernatant was removed, the cells were resuspended in complete culture solution and inoculated in a culture dish with complete culture medium containing 45 mL of Royal Park Memorial Institute (RPMI)-1640 medium, 5 mL of fetal bovine serum (FBS), 50 μL of a mixture of penicillin/streptomycin at a ratio of 1:1000 and 1000 U γ-interferon (γ-IFN). The cells were evenly distributed through gentle shaking and then placed in the culture incubator. The culture conditions were set at 33 °C with 5% CO_2_ under saturated humidity. The medium was changed the next day, and cell growth was observed. When the cell confluence reached 70–80%, the cells were subcultured at a ratio of 1:3. The cells that were cultured at 33 °C were inoculated into a fresh, precoated culture bottle with γ-IFN-free 1640 medium added. Then, the culture bottle was placed into an incubator at 37 °C with 5% CO_2_. Microscopically, the cells proliferated slowly, the cytoplasm extended to the periphery, and a large number of secondary protrusions emerged from the cell body. With 10–14 days of differentiation and maturation, the cells could be used in related experiments. The differentiated and mature podocytes were evenly inoculated into a six-well plate, with 3 mL of medium added to each well at a certain density. Then, 24–36 h later, when the cell confluence reached approximately 80–90%, the cells were synchronized with 8–12-h starvation culture in FBS-free 1640 culture medium. Grouping was conducted as follows: the NG group, with 5.6 mmol/L d-glucose, and the HG group, with 25 mmol/L d-glucose.

### Cell transfection

The primary podocytes and MPC5 cells in the logarithmic growth phase were detached with trypsin, counted, inoculated into a six-well plate, and diluted with Dulbecco’s modified Eagle’s medium for transfection. Following approximately 24 h of growth when the cell confluence reached 100%, the cells were transfected using Lipofectamine 2000 (Invitrogen, Carlsbad, CA, USA). The cells in each group were then triturated, mixed, allowed to stand for 10–20 min, and then incubated in a cell incubator at 37 °C with 5% CO_2_. The groups included the OE-NC (cells transfected with the OE-NC sequence), OE-PVT1 (cells transfected with the OE-PVT1 sequence), sh-NC (cells transfected with sh-NC), sh-FOXA1 (cells transfected with sh-FOXA1), OE-PVT1 + GSK126 (cells co-transfected with OE-PVT1 and EZH2 inhibitor GSK126), sh-PVT1 (cells transfected with sh-PVT1), pcDNA-NC (cells transfected with pcDNA-NC), pcDNA-FOXA1 (cells transfected with pcDNA-FOXA1), sh-PVT1 + pcDNA-FOXA1 (cells co-transfected with sh-PVT1 and pcDNA-FOXA1), and GSK126 (cells transfected with EZH2 inhibitor GSK126) groups.

### Dual-luciferase reporter gene assay

The target gene of PVT1 was analyzed and predicted by the biological prediction website microRNA.org. The FOXA1 3′-untranslated region sequence was amplified by polymerase chain reaction (PCR). Double enzyme digestion was conducted using the *Xho*I and *Not*I enzyme cutting sites, the target fragment was cloned downstream of the luciferase reporter gene pmirGLO (3577193, Promega, Madison, WI, USA), and the carrier was named pFOXA1-wild type (WT). After picking and sequencing the bacteria, the plasmid was purified for future use. Site-directed mutagenesis was carried out on the binding sites of PVT1 and FOXA1, and the pFOXA1-mutation (Mut) vector was constructed. Two kinds of reporter plasmids were transfected into HEK293T cells together with OE-PVT1 and NC. Twenty-four hours after transfection, the cells were lysed and centrifuged at 25,764 × *g* for 1 min. The supernatants were collected. Luciferase activity was determined by a Dual-Luciferase Reporter Assay System (E1910, Promega). Firefly luciferase activity was measured by the addition of 100 μL of firefly luciferase working solution to each cell sample, and Renilla luciferase activity was tested by the addition of 100 μL of Renilla luciferase working solution. The relative luciferase activity was obtained based on those of firefly luciferase and Renilla luciferase.

### Reverse transcription-quantitative PCR

The total RNA in the tissues and cells was extracted using TRIzol (Invitrogen). A NanoDrop 2000 micro ultraviolet (UV) spectrophotometer (1011U, NanoDrop Technologies, Wilmington, DE, USA) was used to determine the ratios of *A*_260_/*A*_230_ and *A*_260_/*A*_280_ for the total concentration and purity of the extracted total RNA. The complementary DNA (cDNA) was generated through reverse transcription according to the instructions of the TaqMan MicroRNA Assays Reverse Transcription Primer (4427975, Applied Biosystems, Carlsbad, CA, USA). The reverse-transcribed cDNA was diluted to 50 ng/μL, the reaction amplification volume was 25 μL, and 2 μL was added every cycle. The reverse transcription was conducted at 37 °C for 30 min and at 85 °C for 5 s. The primers for lncRNA PVT1 and FOXA1 were designed and synthesized by TaKaRa (Dalian, Liaoning, China) (Table 1). An ABI 7500 quantitative PCR instrument (7500, ABI, Oyster Bay, NY, USA) was used for real-time fluorescence quantitative PCR (qPCR) detection. The PCR conditions were as follows: initial denaturation at 95 °C for 10 min, followed by 40 cycles of denaturation at 95 °C for 10 s, annealing at 60 °C for 20 s, and finally, an extension at 72 °C for 20 s. The fluorescent qPCR volume was 20 μL and included 10 μL of SYBR Premix Ex Taq^TM^ II, 0.8 μL of PCR forward primer (10 μM), 0.8 µL of PCR reverse primer (10 µM), 0.4 µL of ROX Reference Dye, 2 μL of cDNA template, and 6 μL of sterilized, distilled water. β-Actin was used as an internal reference according to the equation ΔCt = Ct_target gene_ − Ct _internal reference_, where Ct is the number of amplification cycles when the real-time fluorescence intensity of the reaction reached the set threshold.

**Table 1:** Primer sequence of RT-qPCR

Gene	Primer sequence (5′–3′)
*LncRNA PVT1*	F: AGAATTAAGAGTGTGGGCAC
	R: GAATGCCCTCTTCTTAGGG
*FOXA1*	F: GCAATACTCGCCTTACGGCT
	R: TACACACCTTGGTAGTACGCC
*GAPDH*	F: GGAGCGAGATCCCTCCAAAAT
	R: GGCTGTTGTCATACTTCTCATGG

*RT-qPCR* reverse transcription-quantitative polymerase chain reaction, *LncRNA PVT1* long noncoding RNA plasmacytoma variant translocation 1, *FOXA1* forkhead box A1;,*GAPDH* glyceraldehyde-3-phosphate dehydrogenase, *F* forward, *R* reverse

### Western blot analysis

Radio-immunoprecipitation assay cell lysis buffer (BB-3209, BestBio, Shanghai, China) was employed to lyse and extract the total protein from the cells. The extracted proteins were separated by sodium dodecyl sulfate-polyacrylamide gel electrophoresis (SDS-PAGE) and then transferred onto a polyvinylidene fluoride membrane at a constant voltage of 80 V. Then, the membrane was blocked for 1 h and was incubated at 37 °C for 1 h with the following primary antibodies: rabbit polyclonal antibodies directed against FOXA1 (ab23738, 1:1000), Bcl-2-associated X protein (Bax, ab199677, 1:1000), B cell lymphoma 2 (Bcl-2, ab196495, 1:500), and cleaved caspase-3 (ab49822, 1:1000). All of these antibodies were purchased from Abcam (Cambridge, MA, USA). The membrane was then washed three times with phosphate-buffered saline (PBS) at room temperature (5 min each time), followed by coloration. The relative expression of the target protein was equal to the gray value of the target protein band divided by the gray value of the internal reference band (the internal reference was glyceraldehyde-3-phosphate dehydrogenase (GAPDH)).

### Methylation-specific PCR

The DNA of the cells was treated with hydrogen sulfite and was purified by Wizard Purification Resin (Promega). Following NaOH treatment, the DNA samples were precipitated with ethanol and resuspended in water. The sequences are shown in Table 2. FOXA1 methylation-specific primers (M) and FOXA1 unmethylation-specific primers (U) were purchased from Invitrogen Inc. The methylation-specific PCR (MSP) amplification conditions were as follows: initial denaturation at 94 °C for 5 min, followed by 44 cycles of denaturation at 94 °C for 15 s, annealing at 60 °C for 10 s, and finally, extension at 72 °C for 9 s. The unmethylation-specific PCR (UNMSP) amplification conditions were as follows: initial denaturation at 94 °C for 5 min, followed by 36 cycles of denaturation at 94 °C for 15 s, annealing at 60 °C for 10 s, and extension at 72 °C for 8 s. The product bands were observed with the UV gel apparatus (Bio-Rad Inc., Hercules, CA, USA) after 1.5% agarose gel electrophoresis (Shanghai Sangon Biotech Co., Ltd., Shanghai, China) and GelRed (nucleic acid dye, Biotium Inc., Fremont, CA, USA) staining.

**Table 2:** Primer sequence for methylation-specific PCR

Gene	Primer sequence (5′–3′)
*FOXA1*methylation	F: TTTTATGAATGGTTTGGGTTTTTAC
	R: ACGACTTAACGTACGAATAACTACG
*FOXA1*—nonmethylation	F: TTTATGAATGGTTTGGGTTTTTAT
	R: CAACTTAACATACAAATAACTACACT

*PCR* polymerase chain reaction, *FOXA1* forkhead box A1, *F* forward, *R* reverse

### RNA FISH

A bioinformatics website (http://lncatlas.crg.eu/) was used to obtain the subcellular localization information for lncRNA PVT1. The subcellular localization of lncRNA PVT1 in podocytes was identified using fluorescence in situ hybridization (FISH). Ribo^TM^ lncRNA FISH Probe Mix (Red) (RiboBio Co., Ltd., Guangzhou, Guangdong, China) was utilized according to the instructions. Coverslips were placed into the wells of a six-well plate; the cells were inoculated in the plate with NG (GS 5 mmol/L) and HG (GS 30 mmol/L) and cultured for 1 day until the cell confluence reached approximately 80%. After the cells were washed with PBS, they were fixed with 1 mL of 4% polyformaldehyde at room temperature, followed by treatment with protease K (2 μg/mL), glycine, and phthalide reagents. Then, 250 μL of prehybridization solution was added, followed by incubation of the cells at 42 °C for 1 h. The prehybridization solution was discarded, and 250 μL of hybridization solution containing the probe (300 ng/mL) was added for hybridization at 42 °C overnight. The cells were washed three times with PBS-0.1% Tween 20 (PBST). The 4′,6-diamidino-2-phenylindole (DAPI) stain was diluted by PBST at a ratio of 1:800 and was added to stain the nucleus; then, the cells were inoculated into a 24-well plate to stain the cells for 5 min, followed by three washes with PBST (3 min each time). An anti-fluorescent quenching agent was used to seal the slides, and five different visual fields were observed and photographed under a fluorescence microscope (Olympus, Tokyo, Japan).

### RIP assay

The basic operation steps of the Magna RNA Co-immunoprecipitation (RIP) RNA-Binding Protein Immunoprecipitation Kit (Millipore Corp., Billerica, MA, USA) were as follows: cells were collected using a cell scraper and then washed two times with precooled PBS. The prepared 100 μL of lysis buffer containing protease inhibitors and ribonuclease inhibitors was added for cell lysis on ice for 30 min. Afterwards, the cells were centrifuged at 12,000 × *g* at 4 °C for 30 min, and the supernatant was transferred into a centrifuge tube for further use. A small amount of supernatant was saved and used as the positive control of input. A total of 1 μg corresponding antibodies, namely, mouse anti-EZH2 (ab13537, Abcam) and 10–50 μL of protein A/G beads, were added to the residual supernatant and incubated at 4 °C overnight. After the immunoprecipitation reaction, centrifugation was performed at 4 °C for 5 min at 3000 × *g*, and the supernatant was discarded. The precipitate of the protein A/G beads was washed 3–4 times with 1 mL of lysis buffer. The tubes were centrifuged at 4 °C for 1 min 1000 × *g* after each wash. Finally, 15 μL of 2× SDS sample loading buffer was added and heated in boiling water for 10 min. The related RNA was isolated from the precipitate and then purified. The binding effect between lncRNA PVT1 and DNA methyltransferase was verified using the specific primers for lncRNA PVT1 with the help of reverse transcription-qPCR (RT-qPCR).

### Chromatin immunoprecipitation

The enrichment of EZH2 in the promoter region of the *FOXA1* gene was detected using a Chromatin Immunoprecipitation (ChIP) Kit (Millipore Corp.). When the cell confluence reached 70–80%, the cells were fixed in 1% formaldehyde for 10 min at room temperature to crosslink the intracellular DNA and proteins. Then, the crosslinked DNA and proteins were randomly sheared into appropriately sized fragments by an ultrasonicator for a total of 15 cycles (10 s each cycle at intervals of 10 s). The fragments were centrifuged at 30,237 × *g* at 4 °C; then, the supernatant was collected into three tubes, and the following antibodies were added: positive control antibody (RNA polymerase II), NC antibody (immunoglobulin G (IgG) of normal mouse), and rabbit anti-EZH2 antibody (ab228697, Abcam). The tubes were incubated at 4 °C overnight, and the endogenous DNA–protein complex was precipitated with protein agarose/sepharose. The supernatant was discarded after transient centrifugation, the nonspecific complex was washed, and the crosslinks were broken at 65 °C overnight. The DNA fragment was extracted, purified by phenol/chloroform, and then recycled. The binding of EZH2 and the FOXA1 promoter region was tested using specific primers for the FOXA1 gene promoter region.

### Immunofluorescence assay

On the seventh day of the in vitro culture of podocytes, the coverslip containing the migrated cells was washed three times with PBS (5 min each time). The cells were fixed using 4% polyformaldehyde for 30 min at room temperature and were washed three times with PBS (5 min each time). Following 15 min of treatment with 0.2% Triton X-100 at room temperature, the cells were sealed with 3% bovine serum albumin at 4 °C for 30 min. Next, the cells were incubated with fluorescent primary antibodies directed against FOXA1 (ab23738, 1:1000), Nephrin (ab136894, 1:1000), synaptopodin (ab224491, 1:2500), and podocin (ab97051, 1:100) in a wet box at 4 °C overnight and washed three times with PBS (5 min each time). All antibodies were purchased from Abcam. Then, the cells were incubated with a fluorescent secondary antibody (1:500) for 2 h at room temperature away from light. Then, the cells were washed three times with PBS (5 min each time) and stained with DAPI (ab104139, 1:100, Abcam) for 10 min at room temperature in the dark. PBS washes were repeated three times (5 min each time). The slide was sealed by sealing agents and observed under an inverted fluorescence microscope.

### Flow cytometry

After transfection for 48 h, the cells were detached with ethylene diamine tetraacetic acid-free trypsin and then collected into a flow tube; this was followed by centrifugation and the removal of the supernatant. The cells were washed three times with cold PBS, were centrifuged, and the supernatant was removed. According to the instructions of the Annexin-V-Fluorescein Isothiocyanate (FITC) Cell Apoptosis Detection Kit (APOAF, Sigma, San Francisco, CA, USA), the Annexin-V-FITC/propidium iodide (PI) staining solution was prepared using Annexin-V-FITC, PI, and 4-(2-hydroxyethyl)-1-piperazineëthanesulfonic acid (HEPES) buffer at a ratio of 1:2:50. Then, 100 μL of staining solution was used to resuspend 1 × 10^6^ cells by oscillation and mixing. The cells were then incubated at room temperature for 15 min, 1 mL of HEPES buffer solution was added, and the solutions were oscillated and mixed. Cell apoptosis was analyzed through the detection of FITC and PI fluorescence at 525 and 620 nm, respectively.

### Statistical analysis

The data were analyzed by SPSS 21.0 software package (IBM Corp., Armonk, N.Y., USA), and the mean and standard deviation were calculated. All experiments were repeated at least three times. A *t* test was employed for the comparison between two groups. An unpaired *t* test was used to examine the normality and homoscedasticity of the means, the heterogeneity of variance was corrected using Welch’s test, and the skewness distribution grade data was tested by the nonparametric Mann–Whitney test. The Shapiro–Wilk method was adopted for the data normality test among multiple groups, and one-way analysis of variance (ANOVA) was applied for data analysis. Sidak correction was used for the comparison of mean values, and the skewed distribution data were examined by the nonparametric Kruskal–Wallis *H* test. When *p* value was <0.05, there was a significant difference.

## Results

### LncRNA PVT1 has high expression in DN

**Fig. 1:**
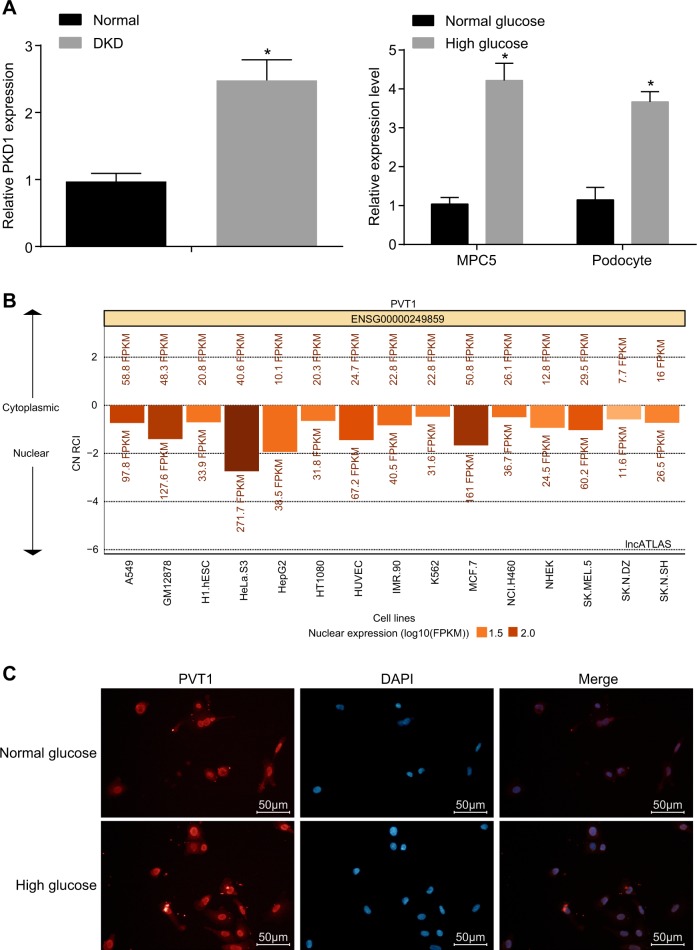
LncRNA PVT1 has high expression in diabetic nephropathy. **a** The expression of lncRNA PVT1 in DN patients, MPC5 cells, and primary podocytes in HG medium as determined by RT-qPCR. **b**, **c** The subcellular location of lncRNA PVT1 was primarily located in the nucleus, predicted by the lncATLAS website and detected by FISH (the original magnification is ×200); *p *<* 0.05 vs. the normal control and the NG group, respectively. The measurement data are expressed as the mean ± standard deviation, and the comparisons between the two groups were analyzed by Student’s *t* test. The experiment was repeated three times. DN, diabetic nephropathy; MPC5, mouse podocyte clone 5; NG, normal glucose; HG, high glucose; DAPI, 4′,6-diamidino-2-phenylindole; PVT1, plasmacytoma variant translocation 1; lncRNA PVT1, long noncoding RNA plasmacytoma variant translocation 1; RT-qPCR, reverse transcription-quantitative polymerase chain reaction; FISH, fluorescence in situ hybridization

Initially, RT-qPCR was performed to determine the expression of lncRNA PVT1, and the results showed (Fig. 1a) that the expression of lncRNA PVT1 in the DN patients (*n* = 32) was significantly higher than that in the normal control (*n* = 26) (*p* < 0.05). Similarly, the PVT1 expression results conducted in MPC5 and primary podocytes suggested that the expression of lncRNA PVT1 in the HG group increased significantly compared with that in the NG group (*p* < 0.05). In addition, the distribution of lncRNA PVT1 in cells was detected by RNA-FISH (Fig. 1b, c), indicating that lncRNA PVT1 was primarily located in the nucleus.

### Downregulation of lncRNA PVT1 inhibits the damage and apoptosis of podocytes induced by HG

**Fig. 2:**
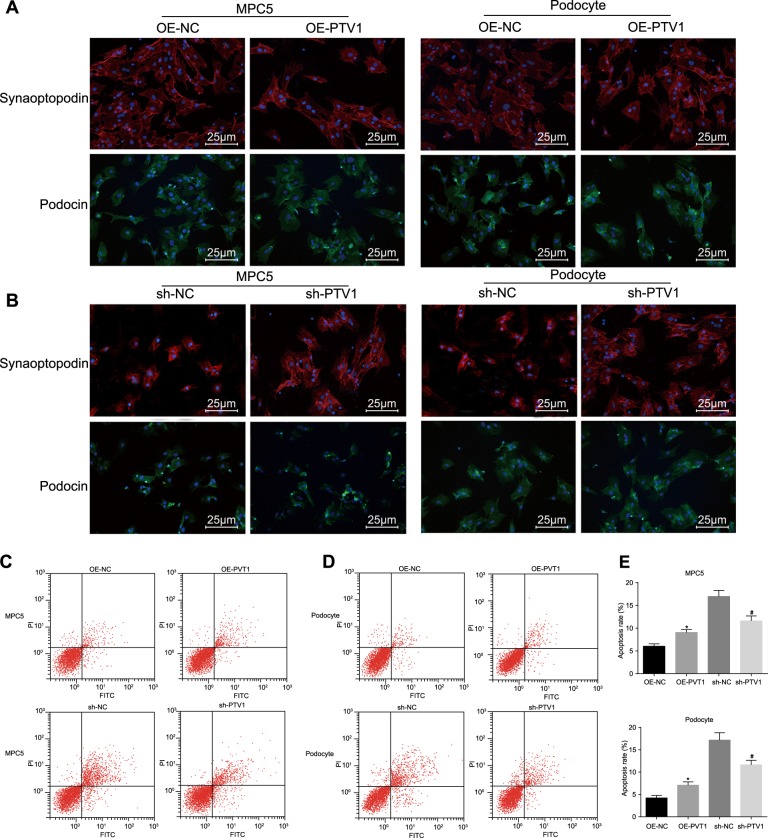
The poor expression of lncRNA PVT1 inhibits the damage to and apoptosis of podocytes. **a**, **b** The cellular morphology of MPC5 and primary podocytes in NG and HG media (the original magnification is ×400). **c**, **d** The apoptosis of MPC5 and primary podocytes in NG and HG media. **e** The apoptosis of MPC5 and primary podocytes in NG and HG media; *p < 0.05 vs. the OE-NC group; ^#^*p* *<* 0.05 vs. the sh-NC group. The measurement data are expressed as the mean ± standard deviation, and the comparisons among multiple groups were analyzed by one-way analysis of variance (ANOVA). The experiment was repeated three times. MPC5, mouse podocyte clone 5; NG, normal glucose; HG, high glucose; OE, overexpressed; NC, negative control; lncRNA, long noncoding RNA; PVT1, plasmacytoma variant translocation 1

The intracellular podocyte marker protein and its localization in each group were observed with immunofluorescence assays. Compared with those in the OE-NC group, the expression levels of synaptopodin and podocin decreased, the filamentous structure of synaptopodin was damaged, and the podocin was crinkled and expressed in the perinuclear area in the OE-PVT1 group (Fig. 2a). Compared with those in the sh-NC group, the expression of synaptopodin and podocin in the sh-PVT1 group was upregulated, the filamentous structure of synaptopodin was recovered, and podocin was redistributed uniformly in the cytoplasm in the sh-PVT1 group (Fig. 2b). The MPC5 and primary podocytes cultured in NG medium were observed, showing that the apoptosis rate of podocytes in the OE-PVT1 group was significantly increased compared with that in the OE-NC group (*p* < 0.05; Fig. 2c, e). The MPC5 and podocytes cultured in HG medium were observed, indicating that the apoptosis rate of cells in the sh-PVT1 group decreased greatly compared with that in the sh-NC group (*p* < 0.05; Fig. 2d, e). The above results indicate that podocytes are damaged in the HG environment, while silencing PVT1 can reduce apoptosis and ameliorate damage to podocytes caused by HG.

### LncRNA PVT1 inhibits the expression of FOXA1

**Fig. 3:**
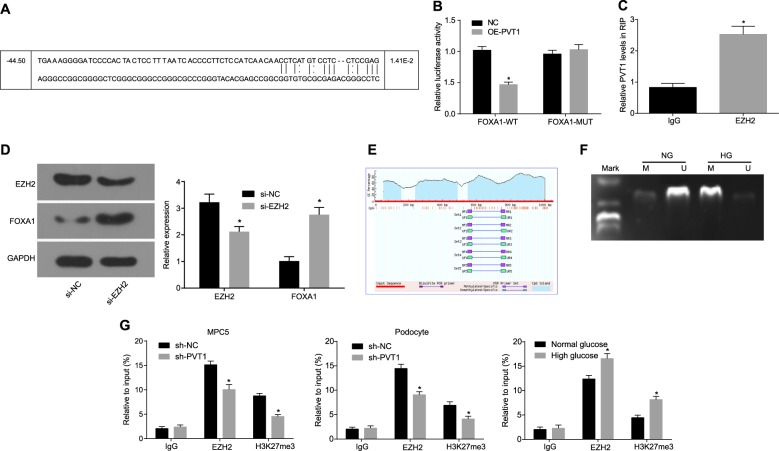
LncRNA PVT1 inhibits the expression of FOXA1. **a** The target binding site between PVT1 and FOXA1 predicted by the biological prediction website microRNA.org. **b** The measurement of luciferase activity to verify the relationship of PVT1 and FOXA1; *p < 0.05 vs. the NC group; **c** the enrichment of IgG and EZH2 by PVT1; *p < 0.05 vs. IgG. **d** The protein levels of EZH2 and FOXA1; *p < 0.05 vs. the si-NC group. **e** The CpG island sites in the FOXA1 promoter region. **f** The methylation levels in cells cultured in HG medium and NG medium as detected by MSP assay. **g** The methylation level of EZH2 and H3K27me3 as detected by ChIP; *p < 0.05 vs. the sh-NC group. The measurement data are expressed as the mean ± standard deviation, and the comparisons between the two groups were analyzed by a *t* test. The experiment was repeated three times. LncRNA PVT1, long noncoding RNA plasmacytoma variant translocation 1; FOXA1, forkhead box A1; CpG, C-phosphate-G; NG, normal glucose; HG, high glucose; EZH2, enhancer of zeste homolog 2; IgG, immunoglobulin G; GAPDH, glyceraldehyde-3-phosphate dehydrogenase; NC, negative control; DN, diabetic nephropathy; MPC5, mouse podocyte clone 5

It was predicted that there was a target binding site between PVT1 and FOXA1 (Fig. 3a), which was further verified by the dual-luciferase reporter gene assay (Fig. 3b). Compared with that in the NC group, the luciferase activity of the FOXA1-WT group decreased remarkably (*p* *<* 0.05). However, the luciferase activity in the FOXA1-MUT group did not change significantly (*p* > 0.05). It was assumed that PVT1 inhibited the expression of FOXA1. Additionally, the RIP assay was employed to determine the binding proteins of PVT1, and the results showed (Fig. 3c) that compared with those in the IgG control, EZH2 was significantly enriched by PVT1 (*p* < 0.05), and the silencing of EZH2 significantly increased FOXA1 expression (*p* < 0.05; Fig. 3d). The results of methylation analysis of the FOXA1 promoter region indicated that (Fig. 3e) there were a large number of C-phosphate-G (CpG) island sites in the FOXA1 promoter region. The MSP assay revealed (Fig. 3f) that the CpG islands of FOXA1 exhibited higher methylation levels in cells cultured in HG medium and lower methylation levels in cells cultured in NG medium. Therefore, the histone methylation pathway was considered. To verify the recruitment of H3K27me3 and EZH2 in the FOXA1 promoter region, ChIP analysis of the FOXA1 promoter region in the MPC5 and primary podocytes cultured in the NG and HG media was conducted (Fig. 3g). Through determination of the expression of H3K27me3 and EZH2 in the enriched products, it was found that the expression of H3K27me3 and EZH2 in the sh-PVT1 group was significantly lower than that in the sh-NC group and that the recruitment level in the HG medium was higher than that in the NG medium (*p* < 0.05). This finding indicated that the methylation level of the CpG island of FOXA1 greatly correlated with the expression of PVT1. All of the aforementioned results have demonstrated that PVT1 promotes the recruitment of H3K27me3 in the FOXA1 promoter region by recruiting EZH2, thereby reducing the expression of FOXA1.

### Silencing of PVT1 or overexpression of FOXA1 inhibits the apoptosis and damage of podocytes in DN

**Fig. 4:**
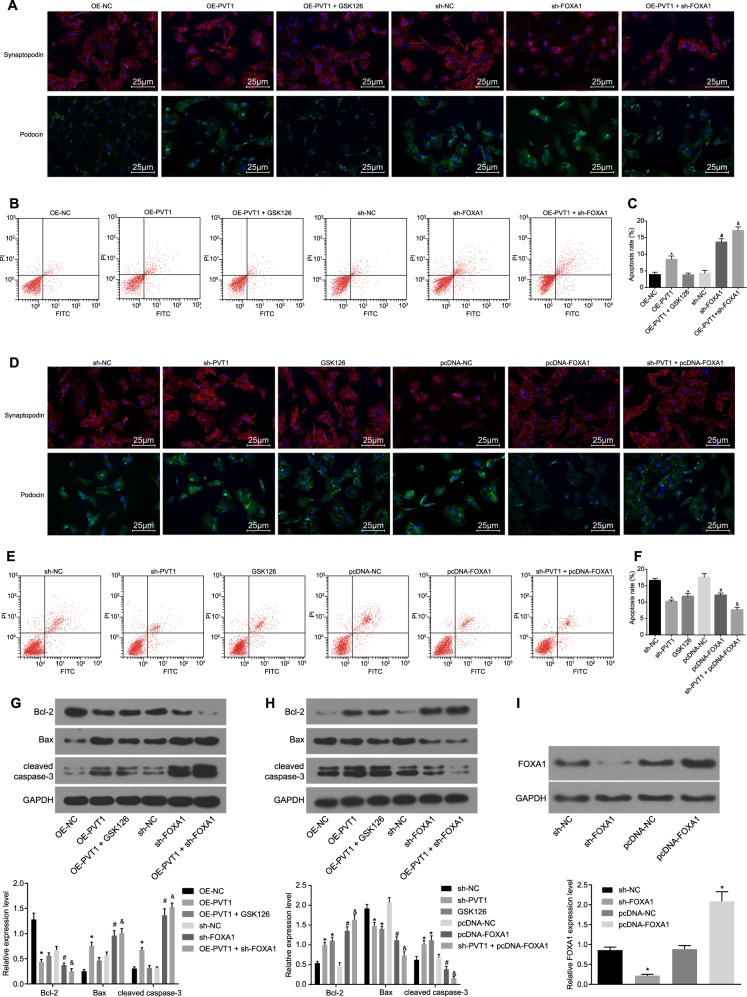
Silencing of PVT1 or overexpression of FOXA1 inhibited the apoptosis and damage of podocytes in DN. **a** Immunofluorescence staining revealing the expression of synaptopodin and podocin in MPC5 cells with normal culture medium (the original magnification is ×400). **b** Flow cytometry map demonstrating MPC5 cell apoptosis conditions with normal culture medium. **c** Apoptosis rate analysis for cells with normal culture medium in response to the treatment of OE-PVT1, OE-PVT1 + GSK126, sh-NC, sh-FOXA1, OE-PVT1 + sh-FOXA1; *p < 0.05 vs. the OE-NC group; ^#^*p* < 0.05 vs. the sh-NC group; ^&^*p* *<* 0.05 vs. the OE-PVT1 group and the sh-FOXA1 group. **d** Immunofluorescence staining revealing the expression of synaptopodin and podocin in MPC5 cells with HG culture medium (×400). **e** Flow cytometry map demonstrating MPC5 cell apoptosis conditions with HG culture medium. **f** Apoptosis rate analysis for cells with HG culture medium in response to the treatment of OE-PVT1, OE-PVT1 + GSK126, sh-NC, sh-FOXA1, and OE-PVT1 + sh-FOXA1. **g** The protein levels of apoptosis-related factors (Bcl-2, Bax, and cleaved caspase-3) in response to the treatment of OE-PVT1, OE-PVT1 + GSK126, sh-NC, sh-FOXA1, and OE-PVT1 + sh-FOXA1 with normal culture medium. **h** The protein levels of apoptosis-related factors (Bcl-2, Bax, and cleaved caspase-3) in response to the treatment of OE-PVT1, OE-PVT1 + GSK126, sh-NC, sh-FOXA1, and OE-PVT1 + sh-FOXA1 with HG culture medium; *p < 0.05 vs. the sh-NC group; ^#^*p* < 0.05 vs. the pcDNA-NC group; ^&^*p* < 0.05 vs. the sh-PVT1 group and the pcDNA-FOXA1 group. I, The protein level of FOXA1 after the silencing or overexpression of FOXA1 in podocytes; *p < 0.05 vs. the sh-NC group; ^#^*p* < 0.05 vs. the pcDNA-NC group. The measurement data are expressed as the mean ± standard deviation, and the comparisons among multiple groups were analyzed by one-way analysis of variance (ANOVA). The experiment was repeated three times. PTV1, plasmacytoma variant translocation 1; DN, diabetic nephropathy; MPC5, mouse podocyte clone 5; HG, high glucose; NC, negative control; OE, overexpressed; GSK126, EZH2 inhibitor; FOXA1, forkhead box A1; Bcl-2, B cell lymphoma 2; Bax, Bcl-2-associated X protein; GAPDH, glyceraldehyde-3-phosphate dehydrogenase

The MPC5 cell lines cultured in NG medium were detected by immunofluorescence assays (Fig. 4a), showing that the expression of synaptopodin and podocin in the OE-PVT1 group was lower than that in the OE-NC group, but there was no significant difference between the OE-NC group and the OE-PVT1 + GSK126 group (*p* > 0.05). The expression of synaptopodin and podocin in the sh-FOXA1 group was downregulated compared with that in the sh-NC group (*p* *<* 0.05); the expression of synaptopodin and podocin in the OE-PVT1 + sh-FOXA1 group was decreased compared with that in the OE-PVT1 group and the sh-FOXA1 group (*p* *<* 0.05). The results of the flow cytometry and western blot analyses (Fig. 4b, c, g, i) showed that compared with that in the OE-NC group, the cell apoptosis rate of the OE-PVT1 group increased significantly, with lower expression of Bcl-2 and higher expression of Bax and cleaved caspase-3; however, there were no significant differences in the cell apoptosis rate and protein expression in the OE-PVT1 + GSK126 group (all *p* > 0.05). Compared with those in the sh-NC group, the cell apoptosis rate in the sh-FOXA1 group increased significantly, the expression of Bcl-2 and FOXA1 decreased evidently, and the expression of Bax and cleaved caspase-3 remarkably increased (all *p* < 0.05). Compared with those in the OE-PVT1 group and the sh-FOXA1 group, the OE-PVT1 + sh-FOXA1 group exhibited a higher cell apoptosis rate, decreased expression of Bcl-2 and increased expression of Bax and cleaved caspase-3 (all *p* < 0.05).

As for the MPC5 cells cultured in HG medium, the results of the immunofluorescence assay (Fig. 4d) showed that compared with that in the sh-NC group, the expression of synaptopodin and podocin in the sh-PVT1 and GSK126 groups was upregulated. Additionally, the expression of synaptopodin and podocin in the pcDNA-FOXA1 group was increased in contrast to that in the pcDNA-NC group (*p* < 0.05), and compared with those in the sh-PVT1 and pcDNA-FOXA1 groups, the expression of synaptopodin and podocin in the sh-PVT1 + pcDNA-FOXA1 group was upregulated (all *p* < 0.05). In addition, the results of the flow cytometry and western blot analyses (Fig. 4e, f, h, i) revealed that compared with those in the sh-NC group, the cell apoptosis rates in the sh-PVT1 group and the GSK126 group reduced significantly, the expression of Bcl-2 increased evidently, and the expression of Bax and cleaved caspase-3 decreased remarkably. Compared with those of the pcDNA-NC group, the pcDNA-FOXA1 group displayed a lower cell apoptosis rate, the expression levels of Bcl-2 and FOXA1 were remarkably elevated, and the expression levels of Bax and cleaved caspase-3 were evidently reduced (all *p* < 0.05). Compared with those in the sh-PVT1 group and the pcDNA-FOXA1 group, the cell apoptosis rates were reduced in the sh-PVT1 + pcDNA-FOXA1 group, which exhibited higher expression of Bcl-2 and lower expression of Bax and cleaved caspase-3 (all *p* < 0.05). These results suggested that the silencing of PVT1 or overexpression of FOXA1 inhibited the apoptosis and damage of podocytes in DN; however, the methyltransferase inhibitor GSK126 can reverse the apoptosis and damage of podocytes induced by the silencing of PVT1 in HG medium.

### Silencing of PVT1 or overexpression of FOXA1 inhibited the apoptosis and damage of podocytes in DN in vivo

**Fig. 5:**
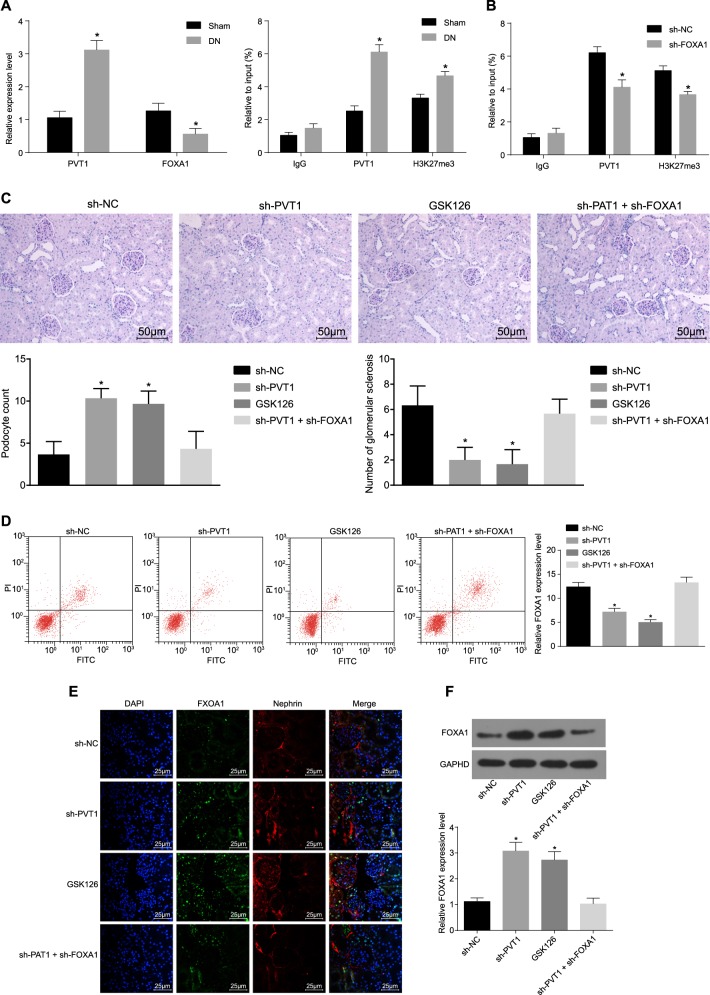
Silencing of PVT1 or the overexpression of FOXA1 inhibited the apoptosis and damage of podocytes in DN in vivo. **a** The expression of PVT1 and FOXA1 in mice with DN in vivo. **b** FOXA1 promoter recruitment in vivo detected by ChIP assay, *n* = 10. **c** The degree of glomerulosclerosis and renal tubule atrophy in response to the treatment of sh-PVT1 and/or GSK126 assessed by periodic acid-Schiff (PAS) staining (the original magnification is ×400). **d** Podocyte apoptosis in response to the treatment of sh-PVT1 and/or GSK126 in vivo. **e** Immunofluorescence staining displaying the expression of podocyte marker Nephrin and FOXA1 in vivo (the original magnification is ×400). **f** The protein level of FOXA1 in vivo detected by western blot analysis; *p < 0.05 vs. the sh-NC group. The measurement data are expressed as the mean ± standard deviation, and the comparisons among multiple groups were analyzed by one-way analysis of variance (ANOVA). The experiment was repeated three times. DN, diabetic nephropathy; PVT1, plasmacytoma variant translocation 1; IgG, immunoglobulin G; FOXA1, forkhead box A1; NC, negative control; DAPI, 4′,6-diamidino-2-phenylindole; GAPDH, glyceraldehyde-3-phosphate dehydrogenase; GSK126, EZH2 inhibitor

To verify whether PVT1 also mediates the damage of podocytes in the same way in vivo, we established a mouse model and established a control group. Compared with those in the control group, the expression of PVT1 in the DN group was significantly higher, and the expression of FOXA1 was remarkably lower (Fig. 5a). The results of the ChIP assay revealed that the recruitment of H3K27me3 and PVT1 in the FOXA1 promoter region of the DN group increased markedly (all *p* < 0.05) (Fig. 5b). After grouping the remaining modeled mice, it was found that compared with those in the sh-NC group, the urine volume, blood sugar level, plasma urea nitrogen, urine protein/urine creatinine ratio, and kidney weight/body weight ratio in the sh-PVT1 and GSK126 groups were evidently lower (all *p* < 0.05), but there were no significant differences regarding each index observed in the sh-PVT1 + sh-FOXA1 group (all *p* > 0.05) (Table 3). Periodic acid-Schiff staining showed that the mesangial matrix was widely proliferated, the mesangial region was broadened, the basement membrane was thickened, and the glomerulosclerosis was slightly hardened in the sh-NC and sh-PVT1 + sh-FOXA groups; the degree of glomerulosclerosis and renal tubule atrophy were significantly relieved in the sh-PVT1 and GSK126 groups (all *p* < 0.05) (Fig. 5c). Compared with those in the sh-NC group, the number of podocytes was elevated and glomerulosclerosis was reduced in the sh-PVT1 group and the GSK126 group (both *p* < 0.05), but there were no significant differences between those of the sh-NC and sh-PVT1 + sh-FOXA1 groups (*p* > 0.05). The results of podocyte apoptosis detection showed that compared with that of the sh-NC group, the apoptosis rate of podocytes in the sh-PVT1 and GSK126 groups decreased significantly (all *p* < 0.05), but did not differ significantly in the sh-PVT1 + sh-FOXA1 group (*p* > 0.05) (Fig. 5d). Podocytes were identified by the podocyte marker Nephrin, and the expression of FOXA1 was determined. The expression of FOXA1 in the sh-PVT1 and GSK126 groups was significantly higher than that in the sh-NC group (all *p* < 0.05), but was not obviously different in the sh-PVT1 + sh-FOXA1 group (*p* > 0.05) (Fig. 5e, f). The above in vivo data suggested that PVT1 could promote the recruitment of H3K27me3 in the FOXA1 promoter region by recruiting EZH2 and inhibiting the expression of FOXA1 to promote the apoptosis and damage of podocytes.

**Table 3:** Comparison of the urine indexes of mice in each group

	sh-NC	sh-PVT1	GSK126	sh-PVT1 + sh-FOXA1
Brine volume (μL)	1475 ± 432	828 ± 516*	799 ± 336*	1535 ± 513
Blood sugar (mmol/L)	22.95 ± 4.76	7.59 ± 1.11*	8.45 ± 1.01*	24.07 ± 5.08
Plasma urea nitrogen (mmol/L)	6.38 ± 1.61	3.65 ± 0.25*	2.92 ± 0.43*	7.25 ± 0.85
Urine protein/urine creatinine ratio	103.27 ± 10.58	31.11 ± 2.13*	34.31 ± 3.82*	110.53 ± 12.66
Renal weight/body weight ratio (mg/g)	12.68 ± 0.97	7.76 ± 0.42*	7.21 ± 0.28*	13.64 ± 1.22

*NC* negative control, *PTV1* plasmacytoma variant translocation 1, *GSK126* EZH2 inhibitor, *FOXA1* forkhead box A1

*p < 0.05 vs. the sh-NC group; *n* = 10

## Discussion

DN, one of the main causes of death, belongs to a clinically common type of disease in microvascular complications^6^. The *PVT1* gene has been revealed to play a role in nephropathy related to type 1 and 2 diabetes^15^. In this study, we aimed to explore the mechanism of lncRNA PVT1 in the development of DN. We revealed that PVT1 promotes the recruitment of H3K27me3 in the FOXA1 promoter region by recruiting EZH2 and thus inhibits the expression of FOXA1 to promote the apoptosis and damage of podocytes in DN, suggesting that downregulation of PVT1 might be a potential target in the treatment of DN.

First, in our study, we observed that the expression of lncRNA PVT1 was significantly higher in DN; the expression of lncRNA PVT1 in MPC5 and podocytes cultured in HG medium was also evidently higher. Similarly, Li et al.^16^ observed that PVT1 presented significantly higher expression in diabetes. In addition, it was found that changes in PVT1 are related to diabetic kidney disease and that glucose can regulate the expression of PVT1 greatly^7^. Our current study also indicates that the overexpression of PVT1 promotes damage and apoptosis of podocytes in DN. Yang et al.^17^ found that the overexpression of PVT1 could promote EZH2 mRNA and protein levels, while the knockdown of PVT1 could inhibit EZH2 expression in vitro and in vivo. Similarly, research conducted by Chen et al.^9^ has elucidated that silenced PVT1 may bind to EZH2 to suppress the expression of microRNA-200c, thus inhibiting the metastasis of melanoma, which might help explain the potential mechanism. As a catalytic core protein in the PRC2, EZH2 stimulates the trimethylation of H3K27me3 and mediates the silencing of the target genes, which participate in senescence, cell differentiation, cell fate decision, cell cycle regulation, cancer formation, and other basic cellular processes^18^. There is evidence demonstrating that EZH2 plays a regulatory role in the expression of cell cycle inhibitors and apoptosis-related genes by mediating H3K27me3 levels^19^. Thus, it is reasonable to infer that lncRNA PVT1 promotes the recruitment of H3K27me3 in the FOXA1 prompter region and reduces the expression of FOXA1 by recruiting EZH2.

Another significant finding of our study was that the silencing of PVT1 or the upregulation of FOXA1 can inhibit the apoptosis and damage of podocytes, while GSK126, an EZH2 inhibitor, can reverse the damage and apoptosis of podocytes in DN. GSK126, a methyltransferase activity inhibitor, has been shown to selectively suppress the activity of EZH2-methyltransferase as well as EZH2 gene expression^20^. It has been reported that PVT1, which is highly expressed in DN podocytes, is induced by HG and is involved in the accumulation of extracellular matrix^7^. GSK126 is a new type of EZH2 inhibitor, and HIC1 recruits EZH2 and DNMT1 to inhibit SIRT1 transcription in response to HG; EZH2 silencing or inhibition (GSK126) relieves SIRT1 repression by HG^21^. In the present study, we observed that the cell apoptosis rates decreased significantly, with evidently increased Bcl-2 expression and decreased expression of Bax and cleaved caspase-3 in the GSK126 group. A previous study demonstrated that preventing podocyte apoptosis could ameliorate renal injury and decrease proteinuria in DN^22^. Oxidative stress has been demonstrated to activate apoptosis by upregulating the expression of tumor growth factor-β and caspase-3 and to disturb the pro-apoptotic–anti-apoptotic (Bax and Bcl-2) balance^23,24^. The oxidative stress in rat kidney tissues was overstimulated, and the imbalance between oxidation and antioxidation aggravated the progress of DN^25^. Bcl-2 has the potential to cause cancer and encodes anti-apoptotic inner mitochondrial membrane protein, and higher Bcl-2 expression has been indicated to be related to the transition to a hormone-unresponsive state with inhibited apoptosis and enhanced proliferation^26^. Caspase-3 is a specific effector caspase for programmed cell death^27^. Key regulators such as Bax, Bcl-2, and cleaved caspase-3 are essential to the intrinsic apoptosis pathway^28^. Zhou et al.^29^ found that PVT1 promotes the expression of Bcl-2, FASN, and CCND1 in osteosarcoma. Ping et al.^28^ found that silencing PVT1 in cisplatin-resistant colorectal cancer cells significantly decreased anti-apoptotic Bcl-2 expression, but increased the expression of pro-apoptotic Bax and cleaved caspase-3^28^. Likewise, Wu et al.^30^ also found that PVT1 knockdown promoted the apoptosis of renal cancer cells, suppressed their proliferation, and increased the expression of cleaved caspase-3. Moreover, the transcription factor FOXA1 is involved in the regulation of apoptosis through the expression of Bcl-2^31^. In this study, the overexpression of FOXA1 increased the expression of Bcl-2 protein and inhibited the apoptosis of DN podocytes.

**Fig. 6:**
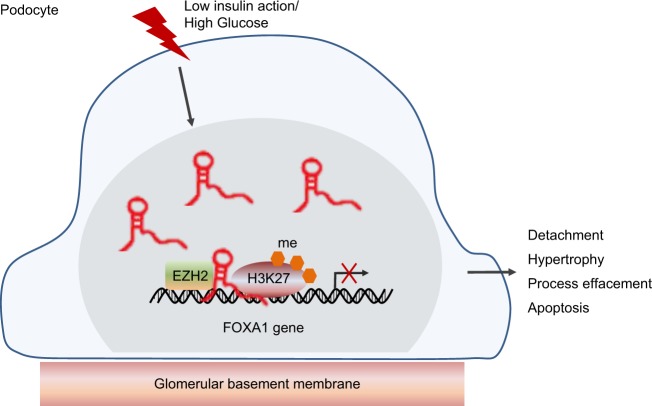
The mechanism map illustrating the regulatory role of lncRNA PVT1 in the damage of podocytes by inhibiting FOXA1. PVT1 can promote the recruitment of H3K27me3 in the FOXA1 promoter region by recruiting EZH2 to inhibit the expression of FOXA1, thereby promoting the damage and apoptosis of podocytes in diabetic nephropathy. LncRNA PVT1, long noncoding RNA; PVT1, plasmacytoma variant translocation 1; FOXA1, forkhead box A1; EZH2, enhancer of zeste homolog 2; H3K27me3, histone 3 lysine 27 trimethylation

To conclude, our findings are important for understanding the etiology of DN and have potential implications for the diagnosis and therapy of DN in relation to the regulatory mechanism of lncRNA PVT1 via FOX1. The results highlight that PVT1 promotes the recruitment of H3K27me3 in the FOXA1 promoter region by recruiting EZH2 and thus inhibits the expression of FOXA1 to promote the apoptosis and damage of podocytes in DN (Fig. 6). However, the sample size of patients is small, and only one cell line was selected in the study; therefore, the results may be unpersuasive to some extent. More research in this field is needed in the future.
